# Management, evolution and risk factors of bile leaks after subtotal cholecystectomies: a cohort study in Quito, Equador

**DOI:** 10.1590/0102-672020260000033e1962

**Published:** 2026-08-17

**Authors:** María de los Ángeles Cela-Sosa, Lizeth Cristina Logacho-Carrera, Walter Iván Díaz-Chamba, Diego Antonio Mena-Noroña, Byron Arturo Montenegro-Muñoz, Franklin Gonzalo Ruales-Rosero, Erika Karina Quishpe-Narváez

**Affiliations:** 1 Pontificia Universidad Católica del Ecuador, Faculty of Medicine – Quito, Equador.; 2 Montenegro & Marcano Cirugía y Estética, Department of Surgery – Quito, Equador.; 3 Hospital General Enrique Garcés, Department of Surgery – Quito, Equador.; 4 Universidade de São Paulo, Faculty of Medicine – Ribeirão Preto (SP), Brazil.

**Keywords:** Cholecystectomy, Biliary Fistula, Choledocholithiasis, Biliary Tract Surgical Procedures, Cholangiopancreatography, Endoscopic Retrograde, Colecistectomia, Fístula Biliar, Coledocolitíase, Procedimentos Cirúrgicos do Sistema Biliar, Colangiopancreatografia Retrógrada Endoscópica

## Abstract

**Background:**

Laparoscopic subtotal cholecystectomy is indicated for severe cholecystitis; however, it may lead to complications, including postoperative bile fistulas.

**Aims:**

To evaluate the management, evolution, and risk factors associated with bile fistulas after laparoscopic subtotal cholecystectomy in a hospital in Quito from January 2019 to June 2022.

**Methods:**

A cross-sectional analytical study with a quantitative approach was conducted. A retrospective review of medical records of patients who underwent laparoscopic subtotal cholecystectomy (n=256) was performed. The dependent variable was the occurrence of bile fistulas, while independent variables included sociodemographic data (age, sex) and clinical factors such as comorbidities, nutritional status, cholecystitis severity (Parkland classification), surgical planning, type of cholecystectomy, anesthetic risk (American Society of Anesthesiologists; ASA I–IV), surgical time, intraoperative complications, time of fistula onset, evolution, and postoperative complications.

**Results:**

The incidence of postoperative fistulas was 12.9% (n=33). Management strategies included drainage (69.7%) and endoscopic retrograde cholangiopancreatography (ERCP) (30.3%). Spontaneous closure occurred in 60.6%, while 30.3% required therapeutic ERCP and 9.1% needed reoperation. Significant risk factors included surgical time >105 minutes (relative risk [RR]: 2.06; 95% confidence interval [CI] 1.04–4.08), type A cholecystectomy (RR 2.19; 95%CI 1.05–4.57), and choledocholithiasis (RR 2.5; 95%CI 1.34–4.67). Logistic regression confirmed surgical time (odds ratio [OR]: 2.6; 95%CI 1.2–5.8) and choledocholithiasis (OR 3.3; 95%CI 1.4–3.9) as significant predictors (p<0.05).

**Conclusions:**

The incidence of postoperative fistulas observed was comparable to previous reports in the literature, highlighting the importance of early identification and appropriate management.

## ARTICLE HIGHLIGHTS

- The incidence of bile leaks after laparoscopic subtotal cholecystectomy was 12.9%.
- Most bile leaks resolved with drainage or therapeutic endoscopic retrograde cholangiopancreatography (ERCP), avoiding reoperation.
- Operative time longer than 105 minutes significantly increased the risk of bile leaks.
- Choledocholithiasis was an independent predictor of postoperative bile leaks.

## Summary

**CENTRAL MESSAGE:**

Type B subtotal cholecystectomies were associated with bile leaks, as were the presence or history of choledocholithiasis and prolonged surgical times, exceeding 105 minutes. Bile leaks were not related to whether a prior ERCP or sphincterotomy were performed, nor to the presence of biliary stents.

**PERSPECTIVES:**

There is currently no standardization regarding the optimal timing for ERCP for bile leaks or the amount of bile leakage that should prompt the procedure. A standardized prospective study could help establish evidence-based management criteria.

## VISUAL ABSTRACT

**Figure:**
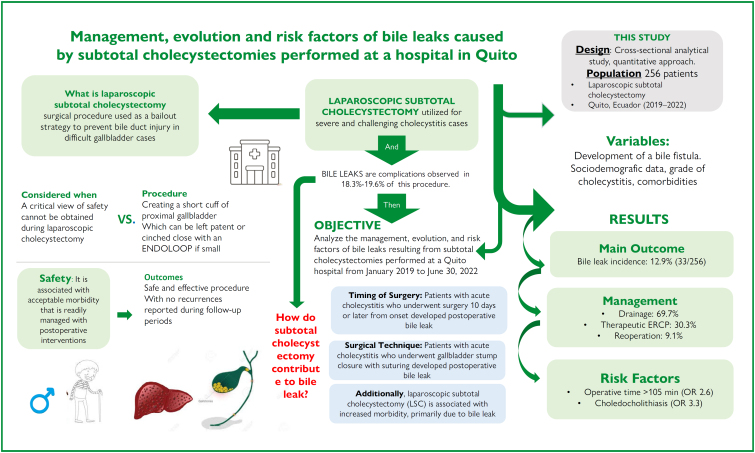

## RESUMO

**Racional:**

A colecistectomia subtotal laparoscópica é um procedimento indicado para casos graves de colecistite; porém, pode apresentar complicações, como fístulas biliares pós-operatórias.

**Objetivos:**

Avaliar o manejo, a evolução e os fatores de risco das fístulas após colecistectomias subtotais laparoscópicas em um hospital de Quito, entre janeiro de 2019 e junho de 2022.

**Métodos:**

Trata-se de estudo analítico transversal, com abordagem quantitativa. Foram analisados retrospectivamente 256 prontuários de pacientes submetidos à colecistectomia subtotal laparoscópica. A variável dependente foi o desenvolvimento de fístula biliar, e as variáveis independentes incluíram dados sociodemográficos (idade, sexo) e clínicos, como comorbidades, estado nutricional, grau de colecistite (classificação Parkland), planejamento cirúrgico, tipo de colecistectomia, risco anestésico (Sociedade Americana de Anestesiologistas; ASA I–IV), tempo operatório, complicações intraoperatórias, tempo de início da fístula, evolução e complicações pós-operatórias.

**Resultados:**

A frequência de fístulas foi de 12,9% (n=33). O manejo incluiu drenagem (69,7%) e colangiopancreatografia retrógrada endoscópica (CPRE) (30,3%). O fechamento espontâneo ocorreu em 60,6%, enquanto 30,3% necessitaram de CPRE terapêutica e 9,1% de reoperação. Fatores de risco associados foram tempo operatório >105 minutos (risco relativo [RR] 2,06; intervalo de confiança [IC] 95% 1,04–4,08), colecistectomia tipo A (RR 2,19; IC95% 1,05–4,57) e coledocolitíase (RR 2,5; IC95% 1,34–4,67). Na regressão logística, tempo operatório (razão de chances [OR] 2,6; IC95% 1,2–5,8) e coledocolitíase (OR 3,3; IC95% 1,4–3,9) foram significativos (p<0,05).

**Conclusões:**

A incidência de fístulas pós-operatórias foi semelhante à encontrada na literatura, ressaltando a importância da identificação precoce e do manejo adequado.

## INTRODUCTION

Laparoscopic subtotal cholecystectomy is a well-established procedure utilized for severe and challenging cholecystitis cases, serving as a bailout strategy to prevent bile duct injury^2,5^. It is associated with acceptable morbidity that is readily managed with postoperative interventions, and it has a low complication rate, with no iatrogenic bile duct injuries reported^2^. Despite its safety profile, it is not without complications, including postoperative fistulas. Therefore, timely detection and treatment are crucial to reduce reoperation rates due to this complication. Additionally, up-to-date information is essential for informed decision-making and for preventing major complications^3,9,11^.

Studies have shown that laparoscopic subtotal cholecystectomy is a safe and effective procedure, with no recurrences reported during follow-up periods^5,8^. The present study reviews bile leaks following subtotal cholecystectomy, a significant complication observed widely reported in the literature. For example, Ibrahim et al.^4^ revealed a 19.6% incidence of bile leakage in acute settings, while Kohga et al.^6^ identified an 18.3% rate in patients with acute cholecystitis.

These findings indicate a notable prevalence of complications related to the surgical technique, underscoring the importance of investigating such occurrences in specific contexts^4,6^. Comparative studies, including that of Lucocq et al.^7^, suggest that subtotal cholecystectomy may be associated with a higher rate of complications than total cholecystectomy. Exploring these differences is crucial for optimizing surgical protocols and enhancing patient outcomes by tailoring strategies to local conditions and resources^7^.

Given the local context in Quito, where subtotal cholecystectomies are performed, and the dearth of prior research in this region, a thorough analysis of bile leak management, evolution, and associated risk factors is warranted. This study aimed to address this knowledge gap and contribute to improving the quality of surgical care.

The objective of this study was to analyze the management, evolution, and risk factors of bile leaks resulting from subtotal cholecystectomies performed at a Quito hospital from January 2019 to June 30, 2022.

## METHODS

### Cross-sectional analytical study, quantitative approach

The study was evaluated and approved by the Ethics Committee for Human Research of Pontificia Universidad Católica del Ecuador (Official letter dated March 10, 2023, No. CEISH-149-2023, Code EO-186-2022). Anonymized information from medical records was authorized for use and provided by the hospital authorities.

The present study was conducted at Hospital General Enrique Garcés, a public institution that serves the southern sector of Quito, the capital of Ecuador. Among the inclusion criteria were: 100% of medical records of patients who were admitted to the surgery room and underwent the subtotal cholecystectomy between January 1, 2019 and June 30, 2022. A total of 2,985 surgical records were reviewed, of which 256 corresponded to subtotal cholecystectomy.

The following were considered exclusion criteria: incomplete or unavailable medical records, histories of patients lost to follow-up, and histories of patients who died in the perioperative period.

The dependent variable was the evolution of bile leak. Among the independent variables, sociodemographic data such as age and sex were considered. Clinical data included comorbidities, nutritional evaluation, grade of cholecystitis (Parkland classification), whether surgery was planned, type of subtotal cholecystectomy, anesthetic risk (American Society of Anesthesiologists; ASA I–IV), surgical time, and intraoperative complications. The following variables were also analyzed: time of onset of the postoperative fistula, clinical course of the fistula, and postoperative complications.

The data were collected retrospectively from the medical records of the follow-up consultations in the outpatient clinic at seven and 30 days.

An Excel sheet developed by the researchers was used to collect data. Once this database was completed and cleaned, it was exported to the Statistical Package for the Social Sciences (SPSS) program, version 25.0, for statistical analysis.

The results were described in univariate form with absolute and relative frequencies. Associations between variables were assessed using the chi-square test and multivariate statistical models.

To avoid selection bias, all eligible medical records were reviewed; to minimize information bias, each medical record was reviewed in its entirety.

The primary outcome was to determine the incidence, management, and evolution of postoperative fistulas in subtotal cholecystectomy patients. The secondary outcomes were to identify the risk factors for postoperative fistula and to relate the ASA anesthetic classification to the management and evolution of these patients.

## RESULTS

A total of 256 records of patients who underwent subtotal cholecystectomy was included. Patients were aged between 15 and 93 years (mean 50.1 years, standard deviation [SD] 16.2 years). The participants’ sociodemographic characteristics are presented in Table 1. Hypertension (10.6%), type 2 diabetes mellitus (3.13%), and other medical conditions (5.86%) were the most frequently reported comorbidities; however, most patients (80,46%) had no comorbidities. In addition, 46.9% of the patients were overweight, and 71.1% had a low anesthetic risk (ASA I).

**Table 1:** Baseline characteristics of the studied population.

Baseline characteristics		Number	%	95%CI
Sex				
	Male	137	53.52	47.20–59.75
	Female	119	46.48	40.25–52.80
Age				
	n=256;			
	Mean: 50.1;			
	SD 16.2;			
	Minimum 15;			
	Q1: 37;			
	Median: 50;			
	Q3: 61;			
	Maximum 93;			
	Mode 51			
Comorbidities				
	No comorbidity	180	70.31	64.31–75.84
	Hypertension	27	10.55	7.07–14.97
	Diabetes mellitus 2	8	3.13	1.36–6.06
	Ischemic heart disease	1	0.39	0.01–2.16
	Cancer	2	0.78	0.09–2.79
	Several comorbidities	15	5.86	3.32–9.48
	Other comorbidities	23	8.98	5.78–13.18
Nutritional evaluation				
	Low weight	2	0.78	0.0009–0.0279
	Normal weight	68	26.56	0.2126–0.3242
	Overweight	120	46.88	0.4063–0.5319
	Obesity grade 1	48	18.75	0.1416–0.2408
	Obesity grade 2	14	5.47	0.0302–0.0901
	Obesity grade 3	4	1.56	0.0043–0.0395
Cholecystitis (Parkland classification)				
	Grade 1	2	0.78	0.09–2.79
	Grade 2	47	18.36	13.81–23.66
	Grade 3	54	21.09	16.26–26.6
	Grade 4	114	44.53	38.34–50.85
	Grade 5	39	15.23	11.06–20.23
Whether surgery was planned				
	Yes	80	31.25	25.62–37.32
	No	176	68.75	62.68–74.38
Type of subtotal cholecystectomy				
	Type A	28	10.94	7.39–15.42
	Type B	228	89.06	84.58–92.61
Anesthetic risk (ASA I–IV)				
	ASA I	182	71.09	65.12–76.57
	ASA II	52	20.31	15.56–25.77
	ASA III	22	8.59	5.46–12.72
Surgical time				
	n=256;			
	Mean 11.6;			
	SD 42.67;			
	Minimum 40;			
	Q1: 79;			
	Median 105;			
	Q3: 135;			
	Maximum 285;			
	Mode 120.			
	>105 minutes	138	53.91	47.59–60.13
	≤105 minutes	118	46.09	39.87–52.4
Intraoperative complications				
	Bleeding	5	1.95	0.64–4.5
	Other	10	3.91	1.89–7.07
	Any	241	94.14	90.52–96.7
Choledocholithiasis				
	No	186	72.66	66.76–78.1
	Yes	70	27.34	21.98–33.2
ERCP pre surgery				
	No	204	79.69	74.23–84.4
	Yes	52	20.31	15.56–25.8
ERCP post–surgery				
	No	236	92.19	88.19–95.2
	Yes	20	7.81	4.84–11.8
Hospitalization days				
	6 days or more	146	57.03	50.72– 63.18
	Less than 6 days	110	42.97%	36.82–49.28
Bile leak				
	No	222	87.06	82.31–90.9
	Yes	33	12.94	9.08–17.7
Time of onset of the postoperative fistula				
	7 days or less	17	51.52	33.54–69.2
	More than 7 days	16	48.48	30.80–66.46
Treatment in bile leak				
	ERCP	10	30.30	15.59–48.7
	Drainage	23	69.70	51.29–84.4
Evolution of bile leak				
	Spontaneous closure	19	57.58	39.22– 74.52
	Needed reintervention	4	12.12%	3.40– 28.20
	Solved by ERCP	10	30.30	15.59–48.71
Other complications				
	Dead by AMI	1	2.63	0.07–13.81
	None	31	81.58	65.67–92.3
	Other	6	15.79	6.02–31.3%
Intervention prescription				
	Biliperitoneum	3	8.57	1.80–23.1
	Not required	31	88.57	73.26–96.8
	Cholestatic syndrome	1	2.86	0.07–14.9

95%CI: 95% confidence interval; SD: standard deviation; Q1: first quartile (25%); Q3: third quartile (75%); ASA: American Society of Anesthesiologists; ERCP: endoscopic retrograde cholangiopancreatography; AMI: acute myocardial infarction.

The incidence of postoperative fistulas in subtotal cholecystectomy patients was 12.94% (33 patients) in three years.

Among those who had postoperative bile fistula, the majority were men (60.6%), were under 65 years old (78.8%), only 30% had comorbidities, and 30.3% were obese. Additionally, 15.2% had a high anesthetic risk (ASA III).

Overall, 75.8% of the patients with postoperative bile fistula had a Parkland classification of grade 3 or higher. Intraoperative complications occurred in 12.1% of cases, and 27.3% had undergone previous ERCP.

In 51.5% of individuals, the fistula appeared before seven days. Regarding management, 69.7% required drainage and 30.3% required ERCP. Spontaneous closure occurred in 60.6% of patients, whereas 30.3% were resolved with ERCP, and 9.1% required reintervention due to bile peritoneum. Other complications occurred in 6.1% of individuals.

The risk factors associated with postoperative fistula were type A subtotal cholecystectomy, operative time longer than 105 minutes, and the presence of choledocholithiasis. Table 2 presents a comparison of the different variables. In a binary logistic regression model, with the presence of postoperative fistula as the dependent variable and the variables that reached statistical significance in the bivariate analysis as cofactors, a certainty of 87.1% was reached, confirming the operative time exceeding 105 minutes and the presence of choledocholithiasis.

**Table 2:** Factors related to the appearance of bile fistula.

Factors		Bile fistula n (%)		RR (95%CI) simple analyze	p-value	OR (95%CI) multivariate analyze	p-value
		Yes	No				
sex							
	Male	20 (60.6)	117 (52.5)	1.33 (0.69–2.56)	0.382		
	Female	13 (39.4)	106 (47.5)				
Age (years)							
	≤64	26 (78.8)	180 (80.7)	1.11 (0.51–2.41)	0.794		
	>65	7 (21.2)	43 (19.3)				
Comorbidities							
	Yes	10 (30.3)	66 (29.6)	1.03 (0.51–2.06)	0.934		
	No	23 (69.7)	157 (70.4)				
	Obesity	10 (30.3)	56 (25.1)	1.25 (0.62–2.49)	0.525		
	Without obesity	23 (69.7)	167 (74.9)				
	ASA III (risk 5%)	5 (15.2)	16 (7.2)	1.99 (0.86–4.63)	0.119		
	ASA I–II (risk 5%)	28 (84.8)	207 (92.8)				
Cholecystitis (Parkland classification)							
	Grade 3 or more	25 (75.8)	182 (81.6)	1.35 (0.65–2.81)	0.425		
	Grade 1–2	8 (24.2)	41 (18.4)				
	Subtotal cholecystectomy Type A	7 (21.2)	21 (9.4)	2.19 (1.05–4.57)	0.043*	0.36 (0.1–1.9)	0.097
	Type B	26 (78.6)	202 (90.6)				
Surgical time (minutes)							
	≤105	22 (66.7)	104 (46.6)	2.06 (1.04–4.08)	0.032*	2.6 (1.2–5.8)	0.004*
	>105	11 (33.3)	119 (53.4)				
Intraoperative complications							
	Yes	4 (12.1)	11 (4.9)	2.22 (0.89–5.48)	0.101		
	No	29 (87.9)	212 (95.1)				
Choledocholithiasis							
	Yes	16 (48.5)	54 (24.2)	2.5 (1.34–4.67)	0.004*	3.3 (1.4–3.9)	0.003*
	No	17 (51.5)	169 (75.4)				
ERCP							
	Yes	9 (27.3)	43 (19.3)	1.47 (0.73–2.97)	0.287		
	No	24 (72.7)	180 (80.7)				

RR: risk ratio; 95%CI: 95% confidence interval; OR: odds ratio; ASA: American Society of Anesthesiologists; ERCP: endoscopic retrograde cholangiopancreatography.

* p<0.05.

No significant association was found between ASA classification and the management of patients with fistulas after subtotal cholecystectomy (p=0.12). Among patients with a higher anesthetic risk (ASA III), 40% required drainage and 60% required therapeutic ERCP, whereas among those with lower anesthetic risk, 75% were managed with drainage and 25% with therapeutic ERCP.

No significant association was found between ASA classification and the evolution of patients with fistulas after subtotal cholecystectomy (p=0.39). Among those with a higher anesthetic risk (ASA III), 40% had spontaneous closure of the fistula and 60% achieved resolution with ERCP. Among those with lower anesthetic risk, spontaneous closure occurred in 64%, ERCP was required in 25%, and reintervention in 10.7%.

## DISCUSSION

The findings of this study on the management and outcomes of postoperative bile fistulas in patients undergoing subtotal cholecystectomy at Enrique Garcés General Hospital from January 2019 to June 2022 provide valuable insights. The incidence of postoperative bile fistulas was 12.9% (33 out of 256 patients), a rate comparable to that reported in prior studies from hospitals with similar case profiles^6,7^. This result highlights the importance of the timely identification and management predisposing factors for this complication.

One of the most significant findings was the identification of factors associated with the occurrence of postoperative bile fistulas. Prolonged operative time (>105 minutes) emerged as a significant risk factor (OR 2.6; 95%CI 1.2–5.8). This finding aligns with existing literature suggesting that longer procedures increase the risk of complications due to prolonged exposure and the handling of inflamed tissues^10^. Extended surgical times may be linked to greater technical challenges, such as adhesions or intraoperative complications. Furthermore, a history of choledocholithiasis was significantly associated with the development of fistulas (OR 3.3; 95%CI 1.4–3.9), underscoring the importance of appropriate preoperative management to minimize risks.

Multivariate analysis confirmed that operative time and the presence of choledocholithiasis were the factors with the greatest statistical significance. These findings are consistent with previous studies, such as that of Ibrahim et al.^4^, which noted that prolonged procedures and the management of complex pathologies increased perioperative morbidity. However, it is important to acknowledge that these factors may be influenced by the clinical condition of the patient and the surgeon's experience, elements that must be carefully considered when evaluating surgical strategies and planning the management of complex cases.

Regarding the management of bile fistulas in the overall cohort, the results showed that most cases were successfully treated with drainage (9,0%) and ERCP (3.9%). Favorable outcomes were characterized by spontaneous closure in 7.8% of patients, reflecting an effective and minimally invasive approach to managing this complication.

These results align with studies such as that by Battal et al.^1^, which highlight ERCP as a safe and effective technique for treating low-grade bile fistulas. Moreover, the use of drainage in low-risk cases and the application of ERCP, when necessary, demonstrate a stepwise approach that reduces associated morbidity.

However, it is worth noting that some cases required surgical reintervention (1.2%), which, although a small percentage, underscores that subtotal cholecystectomy, while effective in avoiding conversion to open surgery, is not without significant risks. This finding aligns with the observations of Lucocq et al.^7^, who noted that postoperative morbidity and the need for reintervention are critical factors to consider when planning surgical treatment for complex cases. The surgical team's experience and a thorough evaluation of preoperative risk factors are key aspects for reducing complications.

A critical limitation of this study was the small number of cases with postoperative fistulas, which hindered the ability to draw definitive associations in specific aspects of their management and outcomes. This limitation underscores the need for additional studies with larger patient populations and diverse hospital settings to strengthen the evidence and allow for more generalizable conclusions. The lack of sufficient data on long-term outcomes also suggests that future research should include extended follow-up to evaluate potential late complications and the efficacy of interventions.

In conclusion, this study provides valuable information on the incidence and management of postoperative bile fistulas in subtotal cholecystectomy, emphasizing the importance of factors such as operative time and a history of choledocholithiasis in the development of these complications. While most fistulas were effectively managed without the need for surgical reintervention, the limited sample size complicates drawing definitive conclusions about outcomes and the best treatment practices. Therefore, the need for further research is evident, focusing on larger study populations and evaluating management methods across different clinical settings. These efforts will contribute to optimizing surgical strategies and improving postoperative outcomes in patients with complex biliary pathologies.

## CONCLUSIONS

Subtotal cholecystectomy, which is a valuable procedure for difficult cholecystectomy cases, has a low likelihood of resulting in bile fistulas. Type A subtotal cholecystectomy is less prone to leakage, whereas the presence of choledocholithiasis and longer surgical time are factors related to the occurrence of leaks. More standardized studies should be conducted to establish the optimal timing and the volume of bile leakage warranting ERCP.

## Data Availability

The datasets generated and/or analyzed during the current study are available from the corresponding author upon reasonable request.
